# Molecular Mechanism of Lipid Accumulation and Metabolism of Oleaginous *Chlorococcum sphacosum* GD from Soil under Salt Stress

**DOI:** 10.3390/ijms22031304

**Published:** 2021-01-28

**Authors:** Hang Su, Jia Feng, Junping Lv, Qi Liu, Fangru Nan, Xudong Liu, Shulian Xie

**Affiliations:** School of Life Science, Shanxi University, Taiyuan 030006, China; 18234115771@139.com (H.S.); fengj@sxu.edu.cn (J.F.); lvjunping024@sxu.edu.cn (J.L.); liuqi@sxu.edu.cn (Q.L.); nanfr@sxu.edu.cn (F.N.); liuxudong@sxu.edu.cn (X.L.)

**Keywords:** *Chlorococcum sphacosum* GD, lipid content, transcriptome sequencing, metabolic pathway

## Abstract

The oleaginous microalgae species *Chlorococcum sphacosum* GD is a promising feedstock for biodiesel production from soil. However, its metabolic mechanism of lipid production remains unclear. In this study, the lipid accumulation and metabolism mechanisms of *Chlorococcum sphacosum* GD were analyzed under salt stress based on transcriptome sequencing. The biomass and lipid content of the alga strain were determined under different NaCl concentrations, and total RNA from fresh cells were isolated and sequenced by HiSeq 2000 high throughput sequencing technology. As the salt concentration increased in culture medium, the algal lipid content increased but the biomass decreased. Following transcriptome sequencing by assembly and splicing, 24,128 unigenes were annotated, with read lengths mostly distributed in the 200–300 bp interval. Statistically significant differentially expressed unigenes were observed in different experimental groups, with 2051 up-regulated genes and 1835 down-regulated genes. The lipid metabolism pathway analysis showed that, under salt stress, gene-related fatty acid biosynthesis (ACCase, KASII, KAR, HAD, FATA) was significantly up-regulated, but some gene-related fatty acid degradation was significantly down-regulated. The comprehensive results showed that salt concentration can affect the lipid accumulation and metabolism of *C. sphacosum* GD, and the lipid accumulation is closely related to the fatty acid synthesis pathway.

## 1. Introduction

With the progressive increase in energy demand worldwide, renewable energy has been used to a clean alternative to replace conventional fuels. Biomass energy is considered to be one of the most promising clean energy sources [1]. Among them, food crops and agricultural waste are evaluated as biomass for first and second generation biofuel production. In particular, oleaginous microalgae are considered to the best third-generation alternative raw materials for the production of biofuels [2,3,4]. More and more oleaginous microalgae species were supposed to be promising feedstock for biodiesel production [5,6]. They can accumulate a large amount of triacylglycerol (TAGs) under appropriate culture conditions to process biodiesel [4,7]. For a long time, researchers have been committed to screening out algae strains with high biomass and lipid content. However, they cannot be directly applied to produce biodiesel at commercial scales. After obtaining an excellent algae strain, it is necessary to further optimize culture conditions and understand mechanism of lipid accumulation [5,8,9].

During the cultivation of algae strains, differences in environmental factors can significantly affect the physiological activities and biochemical composition of cells, such as light, temperature, salinity, etc., directly affecting biomass and lipid accumulation [8,9]. Among them, salinity is one of the most easily controlled factors in the process of algae cultivation, and the economic cost is low [10]. Due to sodium ions in algae cells participating in a series of physiological activities such as acid-base balance adjustment, amino acid utilization, cell membrane permeability changes, etc., it will affect the metabolic pathways of lipid synthesis and accumulation. Therefore, the NaCl concentration in culture medium has an important impact on algal lipid accumulation and lipid quality [10,11]. Kaewkannetra et al. reported the highest lipid content (36%) of *Scenedesmus obliquus* for biodiesel production under 0.3 M NaCl stress conditions, and the algal storage was observed to be approximately 9.5% in the absence of salt [12]. Furthermore, Kaplan et al. believed that the ideal fatty acid composition can be obtained under a suitable salt concentration, such as palmitic acid (C16:0), which reached the highest value of 40.67% of total fatty acids of *Chlorella vulgaris* under 0.9% (*w*/*v*) NaCl, 0.3% (*w*/*v*) glucose, and 0.3% (*w*/*v*) glycerol, or stearic acid (C18:0), which reached the highest value of 22.16% under 2.5% NaCl, 0.6% glucose, and 0.6% glycerol [13]. Thus, the gene expression patterns of algae strains in response to different NaCl concentrations can be analyzed to understand their lipid production mechanisms.

The intracellular lipid metabolism mechanisms of different microalgae are varied; some new species remain unclear. Transcriptome analysis using high-throughput RNA sequencing (RNA Seq) is a powerful tool for studying complex molecular mechanisms. It focuses on the regulation of transcription level in specific physiological states of cells; research from the RNA level does not rely on the full-length genome [14,15,16]. This novel genetic method reduces economic costs while improving sequencing efficiency, which is more conducive to the study of non-model algae species. Reports of transcriptome have involved multiple algae species, usually related to various culture conditions.

The regulation mechanism of the lipid biosynthesis of *Dunaliella parva* under nitrogen deficiency conditions is illuminated based on transcriptome sequencing [17]. Furthermore, the metabolic pathways of astaxanthin biosynthesis for *Haematococcus pluvialis* under photooxidative stress are analyzed by comparative analyses of lipidomes and transcriptomes [18]. Currently, more and more functional genes and molecular mechanisms are disclosed based on transcriptome data in microalgae.

Numerous expressed genes are identified in the lipid biosynthesis in microalgae, which has provided the foundation for illuminating its regulation mechanism. However, the species and physiological conditions are still limited. Previously, a non-model green algae species *Chlorococcum sphacosum* GD, an oil-rich strain isolated from moss by our laboratory, showed outstanding performance in the purification of sewage and lipid accumulation [16,19]. In this study, considering the unique oil accumulation performance under different salt concentrations, the transcriptome analysis of *C. sphacosum* GD under salt stress was carried out by high-throughput sequencing. These different expressed genes provide a foundation for investigation on the molecular mechanism for the lipid biosynthesis of non-model algae strains.

## 2. Results

### 2.1. Biomass and Lipid Content of C. sphacosum GD with Different NaCl Concentrations

**Figure 1:**
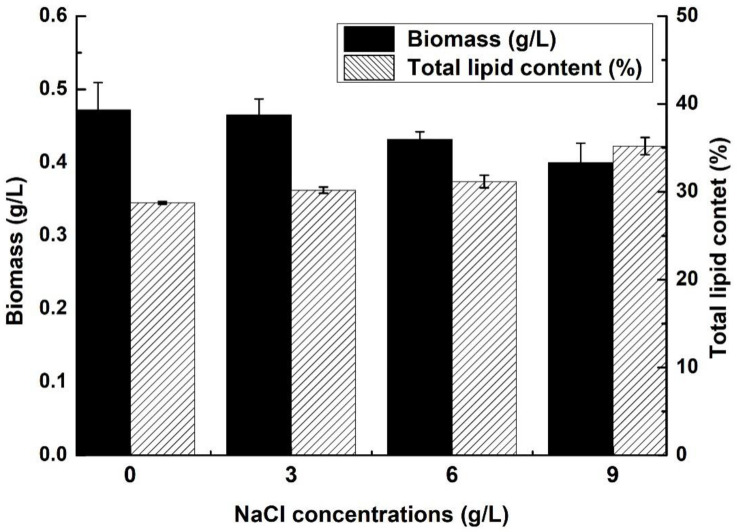
Effects of different NaCl concentrations on cell biomass and total lipid content.

After seven days of salinity culture, algal cells were harvested and measured as shown in Figure 1. The differences were analyzed statistically in terms of biomass and lipid content accumulation of *C. sphacosum* GD from BG11 medium with different NaCl concentrations. In this study, algal dry weight decreased with increasing NaCl concentration in the culture medium: the biomass of the 0 g/L experimental group was above 0.47 g/L, but the 9 g/L experimental group decreased to about 0.40 g/L. However, total lipid content shows the opposite increasing trend, as *C. sphacosum* GD was about 28% without NaCl, but above 35% total lipid content was observed under 9 g/L NaCl concentrations in BG11 medium. The cell biomass was significantly different (F = 4.874, *p* < 0.05) between groups under different NaCl conditions. Moreover, the total lipid content between different NaCl conditions (F = 57.728, *p* < 0.05) was also significant. A possible reason for this phenomenon might be that excessively high NaCl concentration in the growth environment of algae will cause high cell osmotic pressure, which will lead to a decrease in the number of cells; but, in unfavorable environments, total lipid as a source of energy storage in cells will increase its accumulation. Moreover, the tolerance of different algae species to NaCl concentration was different, which leads to different physiological phenomena [11]. Results show that the lipid accumulation ability of *C. sphacosum* GD was relatively improved in the culture environment when increasing the NaCl concentration.

### 2.2. Transcriptome Assembly

**Figure 2:**
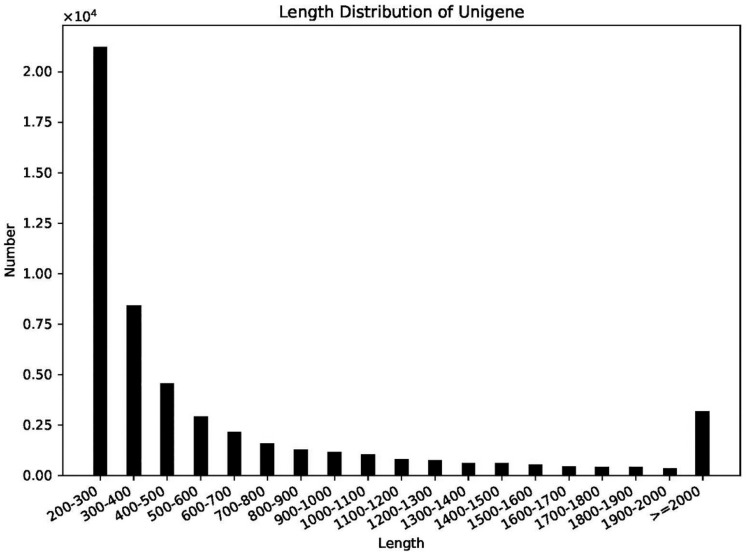
Length distribution of assembled unigenes.

For understanding the lipid accumulation mechanisms in algal cells, we analyzed global changes in gene expression of *C. sphacosum* GD under salt-free and high-salt conditions in artificial medium from transcriptome sequencing (RNA-Seq). Two cDNA libraries prepared from samples cultured in BG11 medium with 0 and 9 g/L NaCl were sequenced repeatedly to produce statistically reliable and comparable RNA-Seq data. After read filtering, 89,674 transcripts were obtained from spliced clean reads, and 52,715 unigenes were obtained from transcript clustering. The unigenes had an average length of 655 bp, maximum length of 11,180 bp, minimum length of 201 bp, N50 of 1071 bp, and N90 of 259 bp. Furthermore, the length distribution of the unigenes is illustrated in Figure 2, with a length of 200 bp–300 bp representing the largest population, accounting for 40.30% (21,243 unigenes).

### 2.3. Gene Functional Annotation

**Figure 3:**
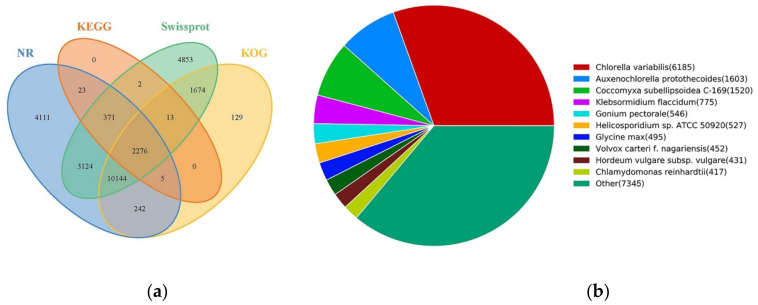
Function annotations of transcripts based on BLAST against diverse databases. (**a**) Venn diagram of transcripts annotated; (**b**) classification of species with gene homology based on NR annotation result.

All unigenes were queried against nine curated databases: CDD (Conserved Domain Database), KOG (euKaryotic Ortholog Groups), NR (NCBI non-redundant protein sequences), NT (NCBI nucleotide sequences), KEGG (Kyoto Encyclopedia of Genes and Genomes), SwissProt (A manually annotated and reviewed protein sequence database), PFAM (Protein family), GO (Gene Ontology), Swiss-Prot (A manually annotated and reviewed protein sequence database) and TrEMBL (European Molecular Biology Laboratory (EMBL) nucleotide sequence), annotated in at least one database of 30,964 unigenes (58.74%), annotated in all database of 1071 unigenes (2.03%), as shown in Table 1. Genes that can be annotated in multiple databases are considered more reliable. The venn diagram of transcripts annotated in NR, KEGG, Swissport, KOG databases shown that 2276 common genes were annotated in Figure 3. Based on the NR annotation result, the related microalgae species with the most homologous genes was *Chlorella variabilis* (6185 genes), followed by *Auxenochlorella protothecoides* (1603 genes), *Coccomyxa subellipsoidea* (1520 genes), *Klebsormidum flaccidum* (775 genes), and others. These results implicate that *C. sphacosum* GD has more related annotation genes consistent with *Chlorella variabilis*.

### 2.4. Gene Function Classification

**Figure 4:**
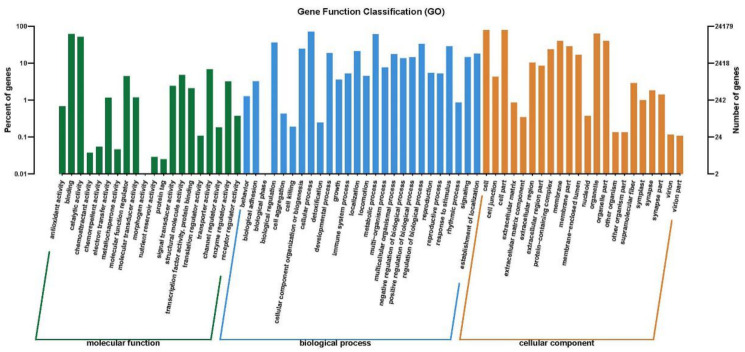
Gene function classification according to GO assignments for the predicted transcripts.

The predicted transcripts of *C. sphacosum* GD were classified according to databases assignments. A total of 219,964 unigenes (45.87%) were assigned at least one GO term, which were classified into three categories of molecular function (56,621, 25.74%), biological process (95,991, 43.64%), and cellular component (66,352, 30.16%), as shown in Figure 4. These genes were further divided into functional subcategories, among which genes corresponding to the “molecular function” group were divided into 20 subcategories, the “biological process” group were divided into 26 subcategories, and the “cellular component” group were divided into 22 subcategories.

**Figure 5:**
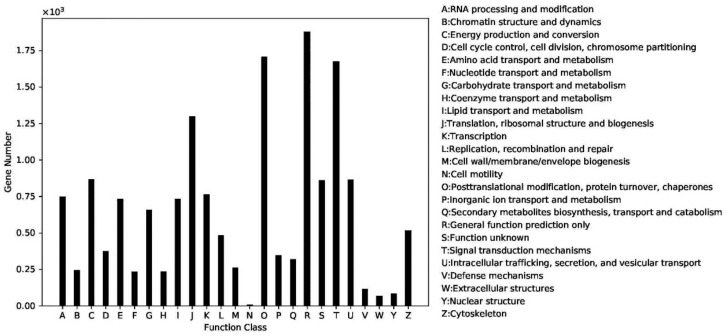
Gene function classification based on the KOG annotation for the predicted transcripts.

A total of 14,483 unigenes were functionally annotated according to KOG assignments, which were classified into 25 categories, as shown in Figure 5. Among these categories, the largest gene number was that of the R group, with predicted function for “general function prediction only”, was 1877; this was followed by the “O” group of genes, which ranked second, and contained 1708 unigenes with predicted function for “Posttranslational modification, protein turnover, chaperones”; the T group contained 1675 unigenes with predicted function for “Signal transduction mechanisms”; the J group contained 1300 unigenes with predicted function for “Translation, ribosomal structure and biogenesis”.

**Figure 6:**
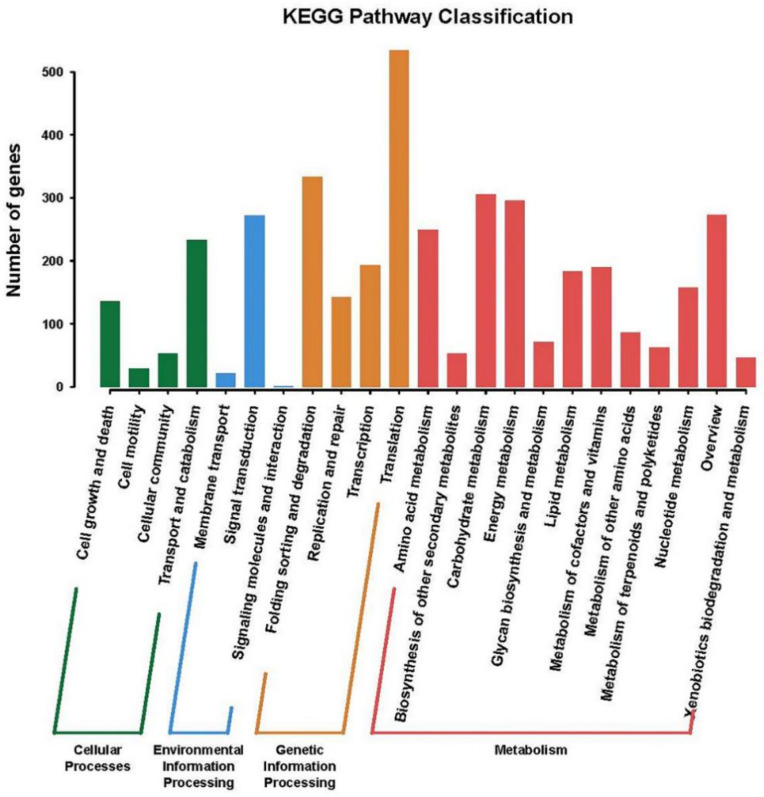
Gene function classification based on the KEGG annotation for the predicted transcripts.

After annotating genes from KO assignments, 3918 unigenes were mapped and clustered to KEGG pathways based on the connection between predicted function and pathway, as shown in Figure 6. They were classified into four categories: “Cellular Processes” was divided into four sub-categories, with 450 genes in total; “Environmental Information Processing” was divided into three subcategories, with a total of 294 genes; “Genetic Information Processing” was divided into four sub-categories with 1203 genes, of which “Translation” accounted for the most with 535; “Metabolism” was divided into 12 subcategories, and the number of genes was, at most, 1971.

### 2.5. Differential Gene Expression Level

**Figure 7:**
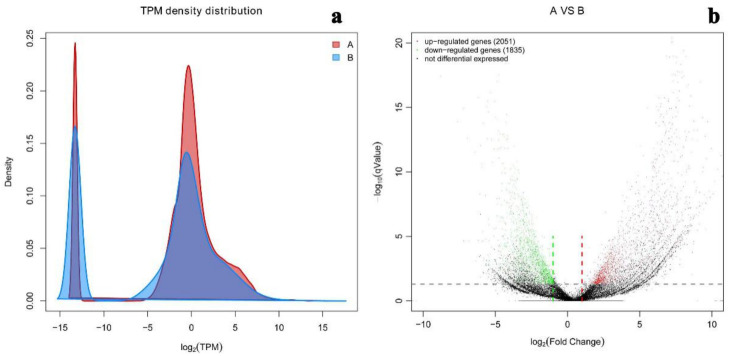
Different gene expression patterns. (**a**) TPM density distribution; (**b**) volcanoplot showing the up and down regulated genes.

Gene expression levels from each experimental condition were counted to determine the transcript abundances as TPM values, with different gene expression patterns observed at different TPM density distributions, as shown in Figure 7a. The calculation results show that unigenes are significantly different in terms of their transcription abundance. DESeq was used to analyze the significantly different genes between the two experimental groups, with screening conditions set to: q value < 0.05 and the multiple of difference |FoldChange| > 2. Differentially expressed genes with statistical significance were observed with 721 up-regulated unigenes and 818 down-regulated unigenes compared two samples under salt-free and high-salt conditions; statistical results of differential genes are shown in Figure 7b. According to the experimental culture conditions, the different gene expressions were mainly caused by the changing NaCl concentration. In the two samples, genes with the same expression level have different densities that reveal a variance of gene expression response to NaCl concentration.

### 2.6. KEGG Enrichment of Differential Gene Expression

**Figure 8:**
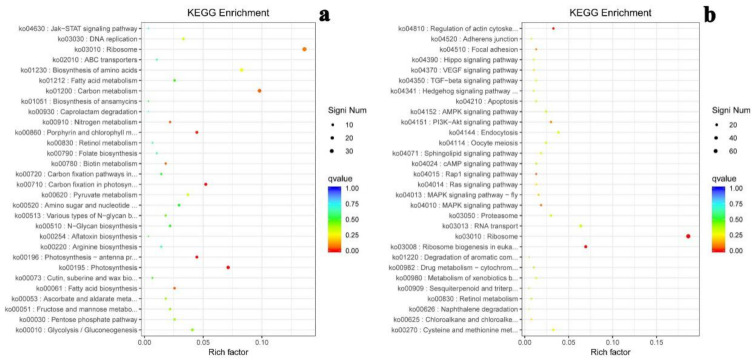
Enriched distribution point diagram of KEGG pathways for significant genes under salt-free condition. (**a**) Enriched pathways for down-regulated genes; (**b**) enriched pathways for up-regulated genes.

From functional gene analysis to metabolic pathway analysis, the enrichment of differentially expressed genes in different metabolic pathways was calculated according to KEGG annotation. The top 30 enriched KEGG pathways involving down-regulated genes and up-regulated genes under salt-free condition are shown Figure 8; differentially expressed genes were considered statistically significant when the Qvalue was smaller than 0.5. The top 15 enriched KEGG pathways with Qvalue below than 0.5 are illustrated in Table 2; these represent important metabolism pathways including energy metabolism, carbohydrate metabolism, amino acid metabolism, metabolism of cofactors and vitamins, etc.

### 2.7. Lipid-Related Metabolic Pathway Analysis

The experimental strain *C. sphacosum* GD was reported as an oleaginous alga with potential for biofuel production; the metabolic pathways of lipids were given further consideration. Based on the enrichment results of the KEGG metabolic pathway, the differentially expressed genes were statistically analyzed to reflect the physiological changes of the experimental strains under salt stress conditions at the molecular level. The purpose of this study was to predict fatty acid biosynthesis (ko00061), fatty acid elongation (ko00062), unsaturated fatty acid biosynthesis (ko01040), and fatty acid degradation (ko00071). The enzymes of the differentially expressed genes were indicated, and input into the KEGG database to identify more information. The high-salt group was used as the standard, and the fold change of the salt-free group was reported, as shown in Table 3, with negative values indicating that it was increased under salt stress. Only 16 genes related to fatty acid synthesis process were identified: seven genes associated with fatty acid biosynthesis, which were all notably up-regulated under high-salt conditions; two genes associated with fatty acid elongation; three genes associated with unsaturated fatty acid biosynthesis; four genes associated with fatty acid degradation, which were all notably down-regulated under high-salt conditions.

**Figure 9:**
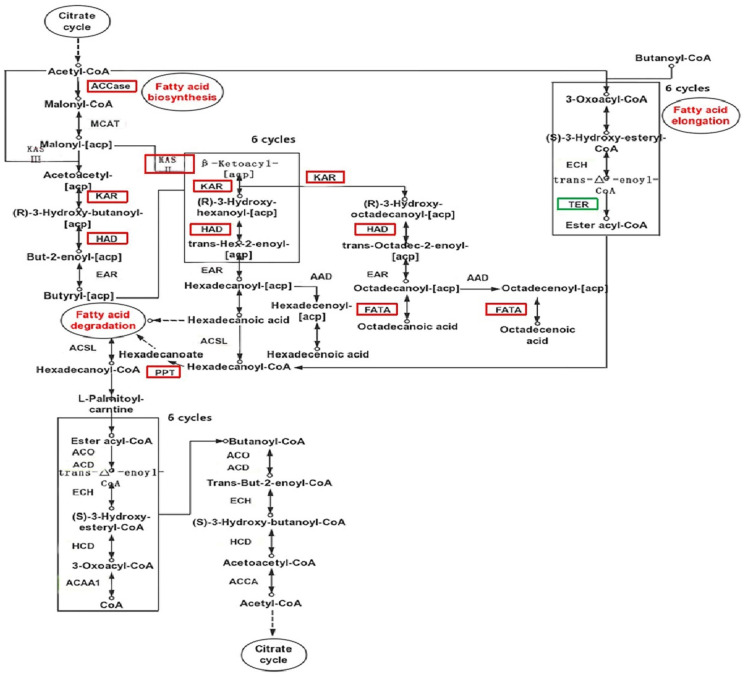
Schematic diagram of fatty acid metabolic pathways. The red boxes represent up-regulated genes under high-salt condition; the green boxes represent down-regulated genes under high-salt condition.

Through the functional annotation of the transcriptome, it is found that the known key enzyme genes are all involved in the fatty acid metabolism of *C. sphacosum* GD. A schematic diagram of the fatty acid metabolic pathways is shown in Figure 9, in which some of the differentially expressed enzymes are labeled. In the process of fatty acid biosynthesis, the first step is to convert acetyl-CoA to malonyl-CoA by the catalysis of acetyl-CoA carboxylase (ACCase). In this study, the gene encoding ACCase (EC: 6.4.1.2) was significantly up-regulated under high-salt conditions. In the second step, malonyl-CoA is transferred to the acyl-carrier protein (ACP) through the action of malonyl-CoA ACP transacylase (MAT, EC: 2.3.1.39). After this step, a round of condensation, reduction, dehydration would be carried out, and then a reduction reaction would again occur, and these would be catalyzed by β-ketoacyl-ACP synthase (KAS), β-ketoacyl-ACP reductase (KAR), β-Hydroxyacyl-ACP dehydratase catalysis (HAD) and enoyl-ACP reductase (EAR), respectively. In this study, all expression of genes encoding KASII (EC: 2.3.1.179), KAR (EC: 1.1.1.100) and HAD (EC: 4.2.1.59) was up-regulated under high-salt conditions. After continuous reduction and dehydration, palmitic acid (C16:0) or stearic acid (C18:0) was finally yielded by the catalysis of acyl—[acyl carrier protein] desaturase (AAD) and fatty acyl-ACP thioesterase A (FATA). Furthermore, the expression of the gene encoding FATA (EC: 3.1.2.14) was up-regulated under high-salt conditions. All the genes encoding ACCase, KASII, KAR, HAD, and FATA involved in fatty acid biosynthesis were all up-regulated in high-salt transcriptional samples. Among them, ACCase is reported to be the key rate-limiting enzyme in the first step, and KASII affects the fatty acid composition as the key enzyme converts C16:0-ATP to C18:0-ATP, so the overall fatty acid synthesis pathway was improved.

Fatty acid elongation begins with stearic acid (C18:0) in mitochondria, which acetyl-CoA provides as a carbon source. The reaction is catalyzed by enoyl-COA hydratase (ECH), trans-2-enoyl CoA reductase (TER) and palmitoyl-protein thioesterase (PPT). After many rounds of condensation, reduction, dehydration and reduction, various long-chain fatty acids are produced. In this study, the expression of the gene encoding PPT (EC: 3.1.2.22) gene expression was up-regulated in high-salt group, but TER (EC: 1.3.1.38) was down-regulated. Many studies have confirmed that TER has a key influence on the synthesis of very long-chain fatty acids, and that PPT participates in the final step of fatty acid formation. Therefore, it is speculated that salt stress would repress the production of long-chain fatty acids, thereby affecting the composition of fatty acids.

Unsaturated fatty acid biosynthesis occurs via the processes of elongation and desaturation with a series of carbon chain fatty acid elongases (FAE) and fatty acid desaturases (FAD). In this study, several desaturation enzymes have also been identified. D9-desaturase (Δ9-SCD, EC: 1.14.19.1) produces oleic acid by desaturating stearic acid with down-regulation, and D12-desaturase (Δ12-FAD, EC: 1.14.19.6) further desaturates oleic acid to form linoleic acid with up-regulation. Their differences indicate that salt-stress plays an important role in the changes in fatty acid composition.

Fatty acid degradation is an oxidation process, which includes β-oxidation and special oxidation methods. At present, special oxidation methods are considered to include propionic acid oxidation, α-oxidation, ω-oxidation and unsaturated fatty acid oxidation. In this study, there was no significant difference in the expression of genes in β-oxidation under different salt conditions. However, ADH5 (alcohol dehydrogenase, EC: 1.1.1.284), ALDH (aldehyde dehydrogenase, EC: 1.2.1.3) and ALDH7a1 (aldehyde dehydrogenase family 7 member A1, EC: 1.2.1.31) occur in α-oxidation and ω-oxidation with down-regulation under high-salt conditions. These data indicate that special oxidation methods of fatty acid degradation may be repressed under salt stress.

Therefore, under high-salt conditions, the fatty acid biosynthesis pathway is up-regulated, but fatty acid degradation is partially inhibited, which explains the observed hyperaccumulation of lipids under salt stress. The coordinated regulation of anabolism and catabolism is an adaptation strategy commonly adopted by microalgae to determine whether products accumulate.

## 3. Discussion

With the continuous advancement of microalgae application research, transcriptomics analysis technology has become a powerful tool for in-depth exploration of microalgae metabolic pathways. Transcriptome sequencing can obtain all transcript data of microalgae at a specific culture period and culture condition [19,20]. Through the analysis of the number and expression abundance of transcripts, the laws of the physiological activities of algae cells, at the level of gene expression, are revealed. Compared with genome sequencing analysis, transcriptome sequencing is a faster and cheaper means of reflecting the dynamic biological process of algae cells with a strong purpose [14,15]. 

In recent years, the transcriptomics analysis of the lipid metabolism pathway in algal cells under different culture conditions has been increasing, which provides a theoretical basis for the modification of the lipid production performance of oleaginous algae strains [21,22,23]. In this study, a transcriptome sample of *C. sphacosum* GD was obtained. There are 52,715 unigenes after de-redundancy, which is similar to the numbers found in other reported strains, such as 33,307 unigenes in *Dunaliella tertiolecta*, 40,916 unigenes in *M.dybowskii* Y2, 50,211 unigenes in *T.* *minus* and 40,916 unigenes in *Navicula* sp. N6. The read lengths of unigenes are mostly distributed in the 200–300 bp range, which is consistent with the reported distribution of some algae unigenes in the 200–500 bp interval. The GC content is higher than 65%; other reported algae strains are mostly around 60%. By analyzing the sequencing data of different algae species, it can be found that the quality of the transcriptome samples is relatively stable; even different algae species in Chlorophyta, Diatoms and Xanthellae do not have large differences in the number of samples.

In third-generation bioenergy research, many scholars believe that oleaginous microalgae have great potential to produce biodiesel. There have been many reports on the lipid production characteristics of a large number of algae strains under different culture conditions [8,9,24,25]. In this study, the biomass and lipid content of *C. sphacosum* GD, under different salt concentrations, were preliminarily determined. Besides, due to the different cultivation methods and cultivation times, such as the lipid content, which was previously reported after 22 days culture, being reported after only 7 days in this study, the harvested algae cells will have certain differences. The amount of lipid accumulated in this study was lower than the value of 40% lipid previously reported by Feng [26]. Overly high NaCl concentrations will change the osmotic pressure regulation of algae cells, adversely affecting the growth of algae cells [22]. Therefore, high NaCl concentration conditions lead to the death of some cells of the experimental algae strain and the decrease in biomass. However, the accumulation of lipid as the energy storage for the cells facing bad conditions increased. Related to the lipid metabolism pathway predicted by the transcriptome, the results showed that the fatty acid biosynthesis pathway-related differential genes in the high-salt group were up-regulated compared with the normal group. In particular, the one-step rate limiting enzyme ACCase was significantly up-regulated. This is consistent with the increase in the lipid content of algae cells. However, the different gene expression levels in the metabolic pathway of fatty acid elongation and unsaturated fatty acid biosynthesis also affect the components of the fatty acid chain, indicating that the response of algal cells to the high salt environment is complex and diverse [27]. The next experiment will continue to explore the impact of key genes on the length of fatty acid chains, through targeted control of gene expression levels to control fatty acid components.

The analysis results of differential gene expression of the transcriptome provide a molecular basis for the study of microalgae lipid accumulation rules to target key genes. The salt concentration affected the differential gene expression levels of fatty acid synthesis in *C. sphacosum* GD, thereby changing the physiological activities of algae cells and the amount of lipid accumulation. In lipid metabolism, the phenomenon of up-regulation and down-regulation of the expression level coexists with salt stress, providing ideas for the next genetic engineering of this algae strain. The editing of key genes for lipid metabolism in microalgae has been reported many times in the model alga *Chlamydomonas reinhardtii*, such as phospholipid: diacylglycerol acyltransferase (PDAT), a key gene in the process of lipid synthesis, for which, after artificially silencing its mRNA, the membrane lipid composition and growth rate of algal cells changed [28]. Acyl-CoA oxidase is a key gene in the process of fatty acid β-oxidation, through artificial interference with this gene reduces the physiological activity of fatty acid degradation, thereby increasing the lipid content in algal cell [29]. Based on the lipid metabolism analysis of this study, the key genes in the process of fatty acid synthesis may have a greater impact on the lipid accumulation of *C. sphacosum* GD. In the follow-up study, the relevant genes can be manually edited to achieve the purpose of engineering algae strains.

## 4. Materials and Methods

### 4.1. Microalga and Culture Conditions

Oleaginous microalga Chlorococcum sphacosum GD was isolated from soil bryophyte by our team in Shanxi University (SXU), and selected for laboratory culture to produce biodiesel [26]. Voucher specimens (No. SAS2013018) were deposited in the herbarium of Shanxi University (SXU), and maintained in a BG11 medium. Cells were cultured in 500 mL flasks with 200 mL BG11 medium, and placed in an orbital shaker with a stirring speed of 200 rpm and constant temperature of 20 °C, under continuous-intensity light of 50 μ mol photons·m^−2^·s^−1^. According to our previous work on culture optimization, NaCI of various concentrations (0 g/L, 3 g/L, 6 g/L, 9 g/L) were added to the BG11 medium to examine the effects of NaCI on the growth of this alga.

### 4.2. Physiological and Biochemical Analyses

After 7 days of culture, the algal cells were collected, and the biomass and lipid content were determined and compared, respectively. Experiments were set as parallel triplicates and repeated twice for validation. SPSS 24.0 software was used for one-way ANOVA statistical analysis.

A quantity of 50 mL of fluid of the cultured algal cell was harvested by filter film with an aperture of 0.45 μm. After drying at 105 °C for 2 h, the dry weights of blank membrane were weighed as DW0; after filtering the algal fluid and drying at 105 °C for 2 h, membranes with microalgal cells were weighed as DW1. The cells’ biomass was calculated according to formula as (DW1–DW0)/50 mL [26].

Total lipid content was determined by gravimetric analysis with via chloroform–methanol extraction. Some microalgae cells were harvested by centrifugation at low temperature and washed with distilled water, then freeze-dried. Dried biomass was extracted with methanol-chloroform (2:1, *v*/*v*) with ultrasonic crushing. Then, the solution was separated by adding chloroform and 1% NaCl solution to make a final volume ratio of chloroform: methanol: water of 1:1:0.9. The chloroform layer was carefully transferred to a vial and evaporated by nitrogen-blowing. The lipid content was calculated gravimetrically [26].

### 4.3. RNA Extraction, cDNA Library Construction and Illumina Sequencing

The NaCl concentrations of 0 g/L and 9 g/L were selected for experimental group algae cells for transcriptome sequencing. There were three technical replicates, and, for specific performance, there were three independent culture units (in different culture containers), samples were harvested and analyzed (*n* = 3, 6 samples in total). After 7 days of culture, 10 mL of fresh algal fluids was harvested with aseptic operation centrifuging for 3 min, 5000 rpm. Then, the supernatant was removed, the algae mud was washed in the centrifuge tube with sterile water, and the above operation was repeated three times. Clean algae cells were obtained in a centrifuge tube to be frozen in liquid nitrogen. Total RNAs of each sample were extracted using the TransZol Plant RNA Kit (FE201-01, Transgen, Beijing, China). DNase I (Takara, Shanghai, China) was added to enzymatically hydrolyze the residual DNA, Oligo (dT) magnetic beads were used to isolate mRNA with poly-(A) structure from the total RNA. The obtained mRNA was broken to short fragments at high temperature in Mg^2+^ reaction buffer. The first strand cDNA was synthesized by adding random hexamer primer and M-MuLV Reverse Transcriptase (RNase H). Second strand cDNA synthesis was subsequently performed using DNA polymerase I and RNase H. Remaining overhangs of purified double-stranded cDNA were blunted via exonuclease/polymerase activities. They were converted into blunt ends via exonuclease/polymerase activities. The 3’end of DNA fragment was adenylated and then PCR amplified. Finally, the PCR products were purified (AMPure XP system, Beckman Coulter, Beverly, CA, USA) and the library quality was evaluated on the Agilent Bioanalyzer 2100 system (Life Technologies, Santa Clara, CA, USA). The TruSeq PE Cluster Kit v3-cBot-HS (Illumina) was used to cluster the index-encoded samples, and the library preparations were sequenced on the Illumina Hiseq platform to generate paired-end reads [15].

### 4.4. RNA-Seq Data Filtering and Analysis

To obtain high-quality clean reads, the raw reads obtained from RNA sequencing were assessed using FastQC, and Trimmomatic was used to remove reads containing adapter, reads containing ploy-N and low-quality reads. The Q20, Q30, GC contents and sequence repeat levels of clean reads were calculated. Transcriptome assembly of clean data was conducted using Trinity, and transcripts was clustered to obtain the unigene. Gene functions were annotated by BLAST searches against the following database: NR (NCBI non-redundant protein sequences), NT (NCBI nucleotide sequences), PFAM (Protein family), Swissprot (UniPort, a manually annotated and reviewed protein sequence), KEGG (Kyoto Encyclopedia of Genes and Genomes Ortholog database), GO (Gene Ontology), TrEMBL (UniPort, the translation of all coding sequences (CDS) in the EMBL nucleotide sequence database) [30].

The gene expression level of each sample was estimated using RSEM. All clean reads were mapped back to the assembled transcriptome to obtain the read count of each gene from the localization results. Using DESeq software to detect the differential expression genes (DEGs) of the two samples, the visual difference analysis results were obtained. The adjusted-*p* < 0.05 and |log2(foldchange)| > 1 were set as the threshold for significantly differential expression to screen out differentially expressed genes under salt stress. Then, all differentially expressed genes were clustered and conducted in the KEGG pathway database to obtain statistical results of the metabolic pathway annotation [31].

## 5. Conclusions

Microalgal cells will undergo different biological changes when facing adverse environmental stresses. The essence of biological changes lies in the changes in their own regulatory mechanisms. The expression levels of key genes in metabolic pathways control changes in lipid content. This study jointly analyzed the significant difference in biomass accumulation and lipid accumulation under salt stress and the expression level of key genes in the lipid metabolism pathway of *C. sphacosum* GD. The analysis results show that the expression levels of different genes in the fatty acid synthesis pathway are inconsistent under salt stress, which provides more feasible directions for the modification of the lipid metabolism pathway in algal cells. This study explored the transcription response of oleaginous algae species to salt stress, and expounded the close correlation between biological activities and metabolic pathways from the perspective of molecular biology, thus providing molecular data for the exploration of microalgae lipid accumulation rules.

## Figures and Tables

**Table 1:** Numbers of transcripts annotated in seven databases.

Database	Number of Genes	Percentage (%)
Annotated in CDD	16,377	31.07
Annotated in KOG	14,483	27.47
Annotated in NR	20,296	38.50
Annotated in NT	16,189	30.71
Annotated in PFAM	11,440	21.70
Annotated in Swiss-Prot	22,457	42.60
Annotated in TrEMBL	20,223	38.36
Annotated in GO	24,179	45.87
Annotated in KEGG	2690	5.10
Annotated in at least one database	30,964	58.74
Annotated in all database	1071	2.03
Total genes	52,715	100.00

**Table 2:** The top 15 enriched pathways of differentially expressed genes in KEGG enrichment.

ID	Pathway Term	Gene Number	Rich Factor	Q-Value
ko03010	Ribosome	102	0.41	0.00000001
ko00196	Photosynthesis—antenna proteins	12	0.52	0.138575956
ko04010	MAPK signaling pathway	9	0.56	0.182751548
ko00710	Carbon fixation in photosynthetic organisms	18	0.40	0.240032566
ko04810	Regulation of actin cytoskeleton	12	0.41	0.279504446
ko04510	Focal adhesion	6	0.60	0.279504446
ko04015	Rap1 signaling pathway	5	0.62	0.279504446
ko03008	Ribosome biogenesis in eukaryotes	26	0.33	0.279504446
ko00830	Retinol metabolism	5	0.62	0.279504446
ko00195	Photosynthesis	21	0.36	0.279504446
ko04151	PI3K-Akt signaling pathway	14	0.38	0.358512098
ko00980	Metabolism of xenobiotics by cytochrome P450	7	0.47	0.375967376
ko00270	Cysteine and methionine metabolism	18	0.35	0.375967376
ko00010	Glycolysis/Gluconeogenesis	22	0.33	0.375967376
ko01220	Degradation of aromatic compounds	3	0.75	0.398007486

**Table 3:** Differential expressed genes involved in fatty acid metabolism pathway under salt-free conditions.

Enzyme	KEGG	Symbol	EC Number	Fold Change
Fatty acid biosynthesis (ko00061)				
acetyl-CoA carboxylase/biotin carboxylase 1	K11262	ACCase	6.4.1.2	−1.25
3-oxoacyl-[acyl-carrier-protein] synthase II	K09458	KASII	2.3.1.179	−2.30
3-oxoacyl-[acyl-carrier protein] reductase	K00059	KAR	1.1.1.100	−1.65
3-hydroxyacyl-dehydratase	K02372	HAD	4.2.1.59	−2.19
fatty acyl-ACP thioesterase A	K10782	FATA	3.1.2.14	−2.01
fatty acid elongation (ko00062)				
palmitoyl-protein thioesterase	K01074	PPT	3.1.2.22	−1.10
mitochondrial enoyl—[acyl-carrier protein] reductase/trans-2-enoyl-CoA	K07512	TER	1.3.1.38	6.74
unsaturated fatty acid biosynthesis (ko01040)				
stearoyl-CoA desaturase (Delta-9 desaturase)	K00507	Δ9-SCD	1.14.19.1	5.30
omega-6 fatty acid desaturase	K10256	Δ12-FAD	1.14.19.6	−1.70
fatty acid degradation (ko00071)				
S-(hydroxymethyl)glutathione dehydrogenase/alcohol dehydrogenase	K00121	ADH5	1.1.1.284	9.01
aldehyde dehydrogenase (NAD+)	K00128	ALDH	1.2.1.3	3.76
aldehyde dehydrogenase family 7 member A1	K14085	ALDH7a1	1.2.1.31	5.94

## Data Availability

Data is contained within the article.
